# Effect of Substrate Pretreatment Process on the Cutting Performance of Diamond-Coated PCB Micro-Milling Tools

**DOI:** 10.3390/mi14010073

**Published:** 2022-12-27

**Authors:** Xiaofan Yang, Huang Li, Haiyang Lin, Yicong Chen, Rongjie Ji

**Affiliations:** 1 College of Marine Equipment and Mechanical Engineering, Jimei University, Xiamen 361021, China; 2 College of Intelligence Science, National University of Defense Technology, Changsha 410073, China; 3 Xiamen Wisdom-Top Digital Manufacturing Technology Institute, Xiamen 361100, China

**Keywords:** PCB milling tool, CVD diamond coating, pretreatment process, tool life

## Abstract

Diamond coatings were deposited on PCB (printed circuit board) carbide milling tool substrates under various schemes of acid and alkali pretreatment by hot filament chemical vapor deposition (HFCVD). Scanning electron microscopy and X-ray coating analysis were used to examine the surface morphology of the milling tools and the impact of de-cobalt from the substrate surface after pretreatment. Milling experiments were carried out to study the cutting performance of diamond-coated PCB micro-milling tools under various pretreatment processes. The results show that abrasive wear, coating flaking, and cutting-edge chipping are the main failure forms of coated PCB milling tools. The substrate pretreatment process with 20 min of alkali etching followed by 20 s of acid etching allows the diamond-coated micro-milling tools to produce the best film–substrate adhesion and substrate strength. These milling tools also have the longest service lives and are suitable for the high-speed cutting processing of PCB.

## 1. Introduction

An essential core part of electronic equipment, a printed circuit board (PCB) performs tasks like a signal exchange and transmission. High-level PCBs, including high-frequency copper-clad boards, packaging substrates, and high-speed multilayer printed circuit boards, are finding increasing use as artificial intelligence and 5G mobile communication technology advance. Copper foil, artificial resin, glass fiber, etc., are all components of a laminated composite material known as PCB. A crucial step in the PCB machining process is the milling of microgrooves. The strong adhesion of the copper layer and the high abrasiveness of the glass fiber during the milling process readily lead to rapid tool wear, chipping, fracture, and other types of failure because of the small size and intricate structure of PCB milling tools. PCB processing is currently facing a significant challenge due to the shorter tool life that results in more frequent tool changes. Therefore, one method for addressing the aforementioned issues is the deposition of high-quality wear-resistant coatings on milling tools [1,2].

Due to its benefits of high hardness, high thermal conductivity, and low friction coefficient, hot filament chemical vapor deposition (HFCVD) diamond coating is a perfect material for PCB tools’ wear-resistant coating since its preparation process is not constrained by the shape of the tool [3]. Because of its excellent thermal hardness and toughness, WC-Co ultrafine-grain cemented carbide is frequently used as the substrate material for micro-cutting tools. When combined with wear-resistant diamond coating, it can significantly increase the service life of micro-milling tools and boost productivity. The tool substrate must be prepared to remove the cobalt on the surface layer before deposition, because the Co-bonded phase of the carbide substrate has a pro-graphitization effect during the diamond growing process, weakening the bond between the coating and the substrate. Additionally, pretreatment can significantly roughen the surface of the tool substrate, boost the mechanical interlocking between diamond and tungsten carbide particles, and improve the coating’s ability to adhere to the substrate [4,5,6]. Over the years, numerous studies have been undertaken on the carbide substrate pretreatment method for CVD diamond-coated tools. To prevent the migration of Co to the diamond interface, Miriam Fischer et al. [7] combined PVD and CVD to deposit a CrN transition layer on the surface of the carbide substrate. Because CrN produces a Cr-C layer during diamond deposition, it can improve the adherence of diamond coatings. However, the process is complex and can impact the uniformity of diamond coatings. Shen et al. [8] pretreated the WC-Co substrate with a combination of sandblasting and acid erosion, which significantly reduced the depth of the brittle de-cobalt layer and enhanced the surface toughness of the substrate. Deng Fuming et al. [9] compared the effects of one-step acid etching and two-step alkali–acid pretreatment methods on the surface morphology of substrate and the adhesion qualities of diamond coatings and found that the two-step approach was more effective. To increase the adherence of diamond coatings, Wang Yong et al. [10] prevented the catalytic graphitization of Co on the surface of WC-Co substrates using boron pretreatment. Currently, acid–alkali two-step chemical pretreatment, the addition of a transition layer, and surface modification treatment are the most widely used techniques for the preparation of carbide tool substrates before CVD diamond deposition. The acid–alkali two-step pretreatment procedure is the most used approach for diamond-coated tool substrate pretreatment in industrial applications due to its low cost, ease of operation, and applicability for carbide tools with complex shapes [11,12,13].

Existing research on the effect of carbide substrate pretreatment on the performance of CVD diamond coating has concentrated mostly on the applications of conventional-sized tools, whereas research on micro-cutting tools has been limited [14]. The typical edge diameter range of milling tools for high-level PCB is 0.5 to 2.5 mm, and the cutting part’s cross-sectional size is small. The lack of a bonding phase brought on by the substrate surface’s de-cobaltization will significantly reduce the cutting edge’s toughness, which in turn will affect the tool’s cutting performance [15]. Therefore, in order not to significantly damage the fracture strength of micro-cutting tools, the optimization of the substrate pretreatment process parameters and the enhancement of the film–substrate bond strength need to be investigated in depth. In this paper, we explored the pretreatment process parameters that can adequately etch the Co on the substrate surface of the milling tool without severely weakening the substrate strength by developing different substrate pretreatment schemes to extend the service life of micro-cutting tools using carbide PCB milling tools as substrate. By using HFCVD technology, a composite coating of microcrystalline and nanocrystals was deposited on the tool substrate by regulating the deposition process parameters of the diamond coating, and a high-performance CVD diamond-coated PCB micro-mill tool with high substrate-fracture strength and strong film–substrate adhesion was prepared. Milling experiments were conducted using IT180 copper-clad laminate (CCL) as the processing material, and the cutting performance of diamond-coated PCB milling tools was investigated by examining the tool life and wear.

## 2. Experimental Materials and Methods

### 2.1. Test Tool

**Figure 1:**
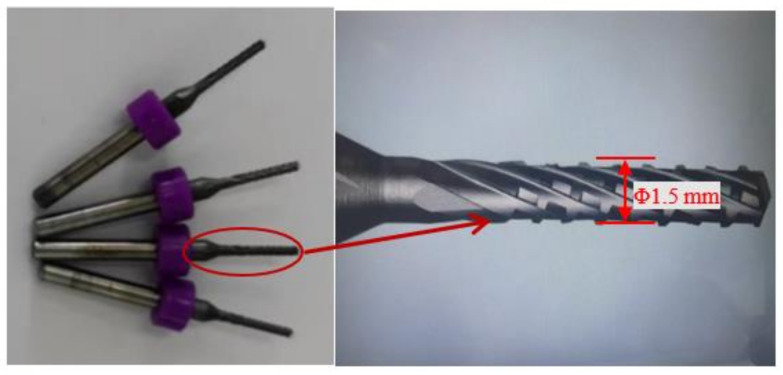
Milling tool used in the test.

A RRC1.5 × 8.5 model PCB carbide milling tool (Xiamen Egret Tools Co., Ltd., Xiamen, China) was used, and the test tool is shown in Figure 1. Tool substrate information is presented in Table 1.

### 2.2. Tool Substrate Surface Pretreatment Scheme

The pretreatment process, which consisted of two acid–alkaline steps, was carried out at 22 °C room temperature. First, the cutting edge portion of the PCB micro-milling tool was immersed in Murakami alkali solution, which has a mass ratio of *m*(KOH):*m*(K_3_[Fe(CN)_6_]):*m*(H_2_O) = 1:1:10. The alkali treatment was used to etch the WC (tungsten carbide) from the carbide substrate’s surface layer and roughen the tool’s surface. The exposed carbide substrate surface’s Co content and surface roughness did not change considerably after the alkali treatment period reached 20 min, based on the results of the prior investigation. Because continuing to extend the alkali treatment duration will weaken the tool substrate further for small-diameter micro-cutting tools, the experimenters decided to carry out acid treatment after alkali treatment for 20 min. The acid solution composition is *V*(H_2_SO_4_): *V*(H_2_O_2_) = 1:10 (volume ratio). Due to the quick pace of the acid reaction, the time required for acid treatment on a carbide substrate is typically between a few seconds and a few minutes. If the acid etching is insufficient, the higher Co content of the substrate surface will lead to the poor bonding of the diamond coating. If the acid etching is excessive, it will lead to the porosity of WC particles on the surface and reduce the toughness of the tool substrate. Four distinct acid treatment time test schemes, A, B, C and D, were chosen in this research to explore the best acid treatment time suitable for PCB micro-milling tools, as shown in Table 2.

### 2.3. Preparation of CVD Diamond-Coated Milling Tool

The preparation of the diamond coatings on the milling tool substrates using HFCVD technology was done for each of the four schemes following the acid–alkaline two-step pretreatment with various schemes. A diamond coating furnace (model: YSL-1) built by Xiamen Xinghongjiye Machinery Manufacturing Co. (Xiamen, China) was used for the coating preparation. CVD coating deposition process parameters was shown in Table 3. The three stages of deposition are nucleation, growth, and the stacking of nanocomposite materials. The diamond film’s crack resistance can be increased, and the coating’s surface roughness can be decreased by using a composite coating structure with the alternate deposition of microcrystalline and nanocrystals. The thickness of the coating was 10 μm, and the coating preparation process took 22 h.

### 2.4. Milling Experiments

The coated milling tools with the four pretreatment schemes, A, B, C and D, were chosen for milling experiments to investigate the impact of various substrate pretreatment schemes on the cutting performance of diamond-coated PCB milling tools. The test milling tools for each scheme in the paper were tested using four milling tools of the same size as a group, and then the test data were obtained by taking the average value. The experiments were carried out using an Anderson forming machine (model: PR-2228/S4), and the workpiece was made of a 6-layer IT180 copper-clad laminate (CCL) with a single layer thickness of 1 mm. Glass-fiber-reinforced resin was used as the layer between the upper and bottom copper foil layers. The experiment processing conditions are shown in Table 4; when the average wear of the side flank of the coated PCB milling tool exceeded 40 μm or the tool was broken, the cutting test was stopped. Following the experiments, an optical microscope (model: VHX2000, Keyence, Esslingen, Japan) and a scanning electron microscope (model: Pure, Phenom, Amstelveen, Netherlands) were chosen to examine the tool’s flank face’s wear morphology and calculate the wear amount VB value.

### 2.5. Testing Instruments

An X-ray coating analyzer (model: XDAL 237 SDD, Fischer, Baden-wurttemberg, Germany), an optical microscope (model: VHX2000, Keyence, Esslingen, Japan), a scanning electron microscope (model: Pure, Phenom, Amstelveen, Netherlands) and a Raman spectroscopy (model: InVia, Renishaw, North East Derbyshire, England) were used to test the experimental results.

## 3. Experimental Results and Analysis

### 3.1. Surface Morphology Analysis of the Substrate of the Milling Tool after Pretreatment

**Figure 2:**
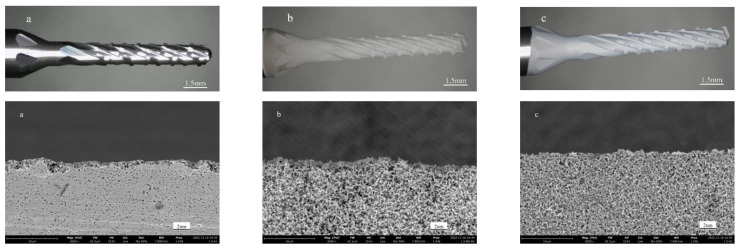
Surface morphology of tool substrate before and after pretreatment. (**a**) Unprocessed; (**b**) Alkali etching for 20 min; (**c**) Acid etching for 10 s.

The cutting part of the carbide tool substrate before and after the acid–alkaline two-step pretreatment is shown in Figure 2, photographed by a light microscope and a scanning electron microscope, respectively. The surface of the untreated tool substrate is flat, as can be seen in Figure 2a. The surface of the substrate exhibits a fluffy coating and becomes rougher after 20 min of alkali etching. Holes appear where the original WC particles were present, and the bonding phase Co is exposed as a result of the reaction of the alkali solution with WC particles and etching. However, Co can encourage the growth of graphite during the deposition of diamond coating, which is not conducive to the nucleation of diamond on the surface of the substrate. At the same time, a significant amount of graphite is formed at the interface between the coating and the substrate, reducing the bonding strength between the film and the substrate. By using acid etching, the Co element can be successfully removed from the substrate’s top layer. As can be observed in Figure 2c, after acid etching pretreatment, the tool substrate surface is obviously roughened with many pits and holes. During the coating preparation process, these low-energy sites are beneficial to promote the nucleation and growth of diamond, while the rough substrate surface can increase the contact area between diamond grains and the substrate surface and enhance the mechanical occlusion effect between the coating and the substrate, thus improving the bonding force between the coating and the substrate [16,17].

### 3.2. Co Content and Depth of De-Cobalt Layer on the Substrate Surface of Milling Tools with Different Pretreatment Schemes

**Figure 3:**
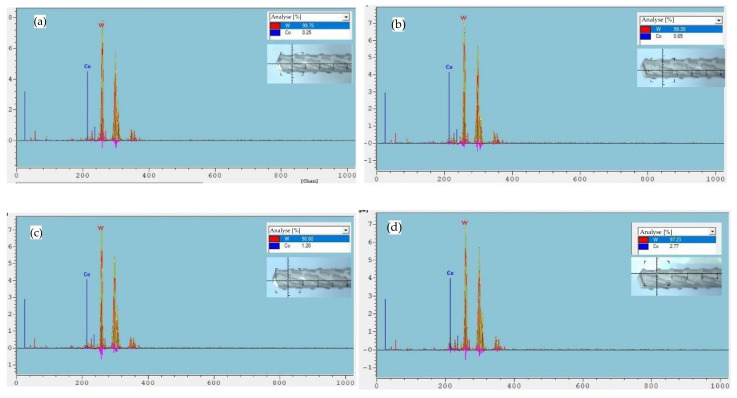
XRF spectra of the substrate surface of milling cutters with different pretreatment schemes. (**a**) Scheme A (Acid etching 30 s); (**b**) Scheme B (Acid etching 20 s); (**c**) Scheme C (Acid etching 15 s); (**d**) Scheme D (Acid etching 10 s).

**Figure 4:**
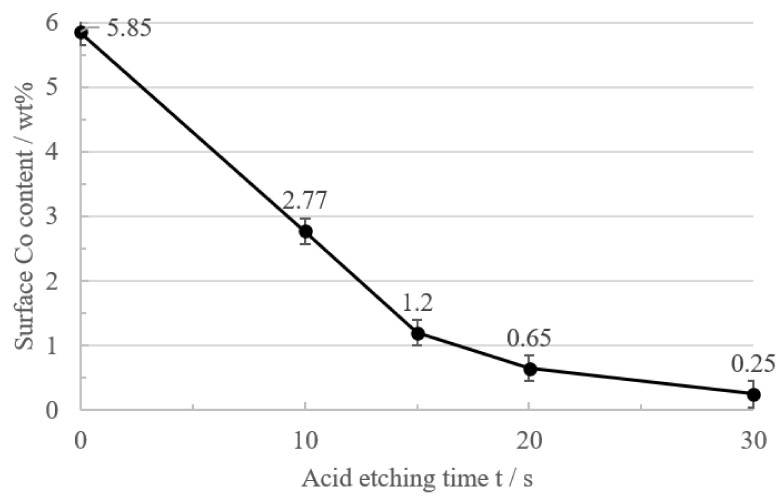
Effect of acid etching time on Co content of cemented carbide surface.

The Co content of the carbide tool substrate surface was detected by the X-ray coating analyzer under different acid etching times, as shown in Figure 3. From the test results, it can be seen that the Co content of the substrate surface in the four pretreatment schemes, A, B, C and D, are 0.25%, 0.65%, 1.20% and 2.77%, respectively. In order to make a more intuitive judgment, the effect of decobaltization under different pretreatment processes was plotted as a graph, as shown in Figure 4. It is evident that the Co concentration on the substrate’s surface gradually declined when the acid etching time was increased. The Co content on the surface noticeably declined in the first 15 s of acid etching, and after 15 s, the rate of decrease steadily slowed. The surface Co concentration dropped to 0.65% after 20 s of acid etching. The substrate’s surface Co concentration was barely 0.25% after 30 s of acid etching.

**Figure 5:**
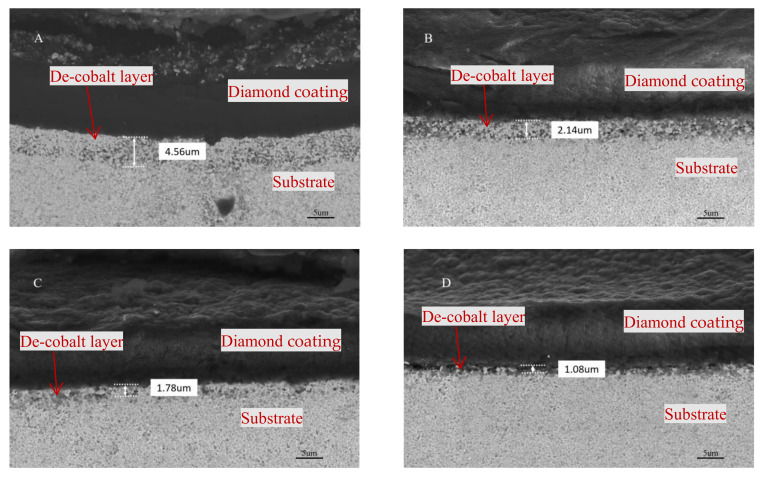
Cross-sectional morphology of milling tools with different pretreatment schemes. (**A**) Scheme A (Acid etching 30 s); (**B**) Scheme B (Acid etching 20 s); (**C**) Scheme C (Acid etching 15 s); (**D**) Scheme D (Acid etching 10 s).

The cross-sectional morphology of a coated milling tool with various pretreatment schemes is depicted in SEM pictures in Figure 5. The cross-section of the milling tool was embedded and polished, and the depth of the de-cobalt layer of the tool substrate was measured. When the acid etching time is 20 s, the depth of de-cobalt layer is about 2.14 μm. This depth increases as the acid etching time increases. When the acid etching duration is increased to 30 s, the de-cobalt layer’s depth quickly rises to 4.56 μm. The fracture strength of the tool will decrease as the depth of the de-cobalt layer increases.

**Figure 6:**
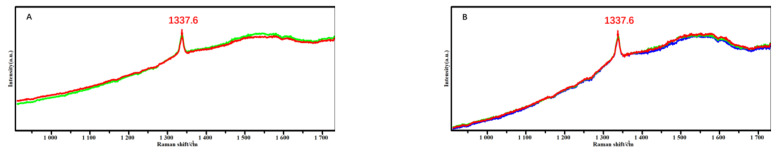

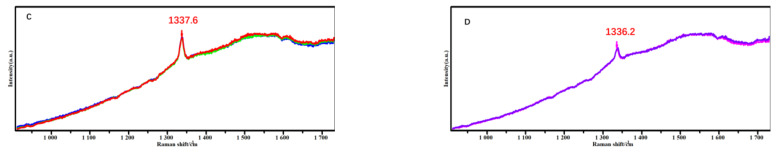
Raman spectra of diamond coatings on milling tools with different pretreatment schemes. (**A**) Scheme A (Acid etching 30 s); (**B**) Scheme B (Acid etching 20 s); (**C**) Scheme C (Acid etching 15 s); (**D**) Scheme D (Acid etching 10 s).

Figure 6 shows the Raman spectra of diamond coatings of milling tools with different pretreatment schemes, and three measurement points were taken on top of the cutting part of each milling tool. From the figure, it can be seen that the positions of the characteristic peaks of diamond SP^3^ phase in the four pretreatment schemes are 1337.6, 1337.6, 1337.6 and 1336.2 cm^−1^ and that the weak peaks of graphite SP^2^ phase broadening near 1580 cm^−1^ are few and almost negligible. Usually, when the diamond and graphite content is the same, the intensity of graphite in Raman spectra obtained under the action of visible light is more than 60 times stronger than that of diamond, so it can be judged that the diamond coating deposited in this experiment is of higher purity. In order to calculate the residual stresses present in the diamond coatings of the four schemes, the following equation can be used [18].

(1) $$\sigma =-0.567\times (v_{m}-v_{0})$$

*v*_m_ is the Raman shift of the SP^3^ carbon structure peak measured by Raman spectroscopy, and *v*_0_ is the Raman shift of the unstressed diamond (1332.5 cm^−1^). The residual stresses of the diamond coatings were calculated to be −2.89 GPa and −2.09 GPa for the four pretreatment schemes, indicating the existence of a certain compressive stress inside the coatings, which is beneficial to improving the fracture strength of the coatings.

### 3.3. Comparative Analysis of the Life of Milling Tools with Different Pretreatment Schemes

**Figure 7:**
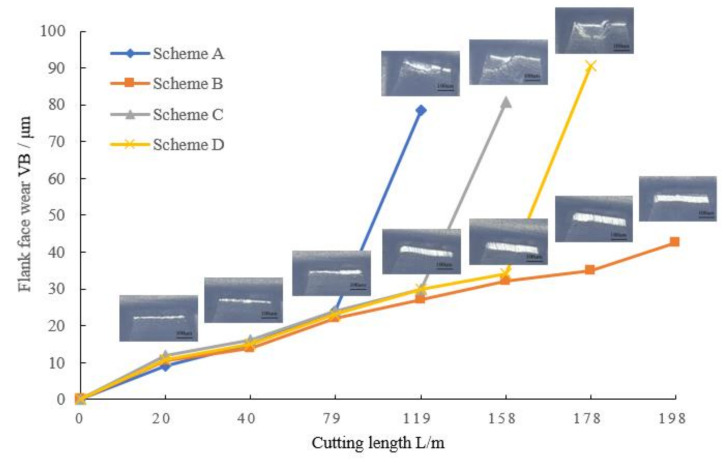
Wear curve about flank face of circumferential edge.

Figure 7 compares the wear curves of diamond-coated milling tools with four different pretreatment schemes under the same cutting conditions. Up until a cutting length of 79 m, the milling tools’ circumferential edge wear values are essentially the same. The milling tools of schemes A, C and D have varying degrees of coating flaking after cutting lengths of 119 m, 158 m and 178 m, respectively. This leads to an increase in cutting resistance and tool failure. When the milling tool of scheme B is cut to 198 m, the flank is worn evenly, and no obvious coating flaking and blade breakage are observed. However, the wear amount is 42.5 μm, which has reached the abrasion standard. It is clear that scheme B has the coated milling tool’s longest useful life. The findings of the cutting test demonstrate that the PCB diamond-coated milling tool is vulnerable to chip blockage and tool breakage under normal wear conditions when the wear amount VB of the flank face reaches 40 μm or higher.

### 3.4. Wear Morphology Analysis of Milling Tools with Different Pretreatment Schemes

**Figure 8:**
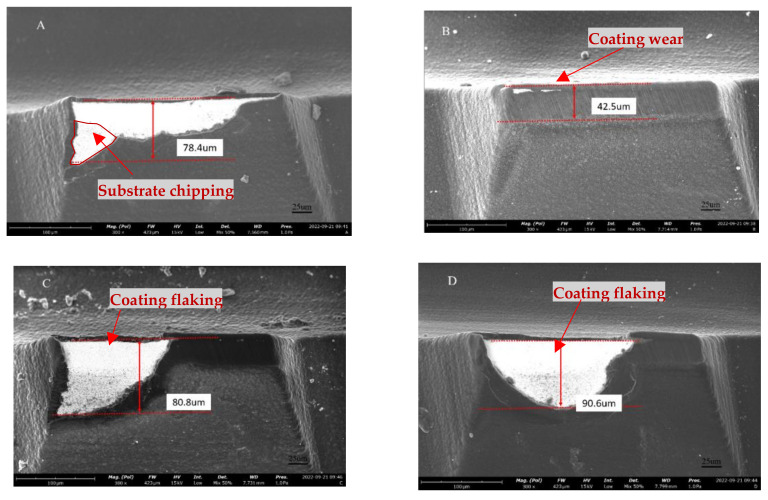
Wear morphology of the flank face of the milling tool with different pretreatment schemes. (**A**) Scheme A (Cutting length L = 119m); (**B**) Scheme B (Cutting length L = 198m); (**C**) Scheme C (Cutting length L = 158m); (**D**) Scheme D (Cutting length L = 178m).

Figure 8 displays SEM images for the wear on the side flank of the diamond-coated PCB milling tool under four distinct pretreatment schemes. It can be seen that, at a cutting length of 198 m, the flank face of the circumferential edge of the scheme B milling tool shows a uniform and flat wear zone. The edge is blunted, and the wear region shows the abrasive wear marks left by the glass fiber hard chip slip. The small part of the coating near the cutting edge has been completely worn off, revealing the white carbide substrate together with the cutting, but there is no coating, flaking or cutting edge chipping on the cutting tool surface, indicating that the coating of scheme B has good film-based bonding and substrate fracture strength. The milling tool of scheme A has a chipped edge with coating and substrate when cutting to 119 m, indicating that the tool substrate fracture strength is insufficient. When the milling tools of schemes C and D, cut to 158 m and 178 m, respectively, both showed extensive coating flaking, and no obvious chipping was found on the exposed carbide substrate, indicating that the adhesion strength of the coating was poor.

Due to the small diameter of micro-cutting tools, varied acid etching times during pretreatment have a substantial impact on the tool substrate strength, as well as the surface adhesion of the diamond coating. As the acid etching duration increases, the Co concentration on the surface of the tool substrate diminishes, and the de-cobalt layer becomes deeper. As the presence of surface Co encourages the formation of graphite in the deposition process, a non-diamond layer forms between the coating and the substrate, thereby reducing the coating’s adhesion strength. The coating’s adhesion decreases as its Co content increases. However, the Co removal produces a brittle WC hard-phase surface layer, which reduces the carbide’s toughness, weakens the substrate’s fracture strength, and makes the tool susceptible to chipping and fracturing. Therefore, the micro-cutting tool substrate surface should include as little cobalt as possible without significantly compromising fracture strength. The test results indicate that, so as to etch Co from the surface of the micro-milling tool carbide substrate without significantly reducing the substrate’s strength, the optimal acid treatment time should be 20 s, based on an alkali treatment of 20 min in conjunction with the CVD diamond coating deposition conditions.

## 4. Conclusions

The diameter of a PCB diamond-coated milling tool’s cutting portion is very small, and the pretreatment process of various schemes will have a significant effect on the adhesion of the diamond coating and the fracture strength of the tool substrate, thereby affecting the tool’s service life.

(1) Four different acid etching times were used for acid and alkali two-step pretreatment on the carbide PCB milling tool substrate, followed by the deposition of CVD diamond coatings. By analyzing the effect of de-cobalt from the substrate surface layer, it was found that the surface Co content had decreased to 0.65% when the acid etching time reached 20 s. Continuing to extend the acid etching time, the surface Co content decreases not obviously, but the depth of the de-cobalt layer will increase rapidly and reduce the fracture strength of the tool.

(2) Experiments on PCB milling were conducted under identical milling conditions, and the results demonstrated that abrasive wear, coating flaking, and cutting-edge chipping are the most common causes of failure for PCB diamond-coated milling tools. The pretreatment technique of acid etching for 20 s after 20 min of alkali etching can achieve the optimal coating film–substrate adhesion and tool substrate strength. Then the milling tool obtains the longest service life.

The objective of the research is to improve the service life of CVD-coated carbide micro tools with complex shapes and to explore ways to reduce the residual stresses inside the coating and enhance the coating–substrate adhesion by optimizing the deposition process parameters of the coating.

## Figures and Tables

**Table 1:** Parameters of the tool substrate.

Tool Specifications (mm) (Blade Diameter × Blade Length × Full Length)	Tool Substrate Material (Average Grain Size 0.8 μm)	Tool Parameters			
		Number of Teeth	Spiral Angle	Circumferential Rake Angle	Circumferential Relief Angle
1.5 × 8.5 × 38.1	6%Co, remaining WC	7	25° (Right)	−3°	15°

**Table 2:** Tool substrate surface pre-treatment program.

Scheme	A	B	C	D
Acid treatment time/s	30	20	15	10

**Table 3:** CVD coating deposition process parameters.

Deposition Parameters	Nucleation Stage	Growth Stage	Eight Alternating Deposition Cycles of Coarse and Fine Grains	
			Microcrystalline	Nanocrystals
Hydrogen flow (mL/min)	2000	2000	2000	2000
Methane flow (mL/min)	50	50	30	88
Reaction pressure (mbar)	3	4	4	4
Filament current (A)	180	185	185	185
Chamber temperature (°C)	750–800	800–850	800–850	800–850
Filament temperature (°C)	1900–2000	1900–2100	1900–2100	1900–2100
Deposition time (h)	1	5	1	1
Distance between hot wire and tool (mm)	20–25	20–25	20–25	20–25

**Table 4:** Milling conditions of PCB milling tool.

Spindle Speed (r/min)	Feeding Speed (mm/s)	Cutting Depth (mm)	Cutting Width (mm)	Cutting Method	Cooling Method
36 × 10^3^	10	6	1.5	Groove milling	Air-cooled
